# Are All Mutations the Same? A Rare Case Report of Coexisting Mutually Exclusive KRAS and BRAF Mutations in a Patient with Metastatic Colon Adenocarcinoma

**DOI:** 10.1155/2017/2321052

**Published:** 2017-07-24

**Authors:** Anusha Vittal, Akshay Middinti, Anup Kasi Loknath Kumar

**Affiliations:** ^1^Department of Internal Medicine, University of Kansas Medical Center, Kansas City, KS, USA; ^2^Division of Medical Oncology, University of Kansas Medical Center, Kansas City, KS, USA

## Abstract

29-year-old Hispanic woman presented to the clinic with complaints of abdominal pain, nausea, fatigue, and constipation. Laboratory tests indicated the presence of iron deficiency anemia and transaminitis. Imaging evaluation revealed marked hepatomegaly with multiple hepatic metastases and pelvic lymphadenopathy. Biopsy of the hepatic lesions showed adenocarcinoma positive for pan-cytokeratin, CMA5.2, villin, and CDX2. She was positive for tumor markers CA 19-9, CA-125, and CEA. Upon further evaluation, she was found to have colorectal cancer positive for KRAS and BRAF mutations. Unfortunately, her disease progressed rapidly and she expired within 3 months from the time of her first diagnosis. KRAS and BRAF mutations are rare enough to be considered virtually mutually exclusive but coexistent mutations appear to be a distinct molecular and clinical subset with aggressive course of illness, which is in dire need of new treatment strategies. Panitumumab and Cetuximab are approved for patients with wild type KRAS CRC. Vemurafenib is a potent inhibitor of the kinase domain in mutant BRAF and its use in BRAF mutated colon cancer remains to be well established. Our report highlights the need to obtain tissue samples from these patients for analysis and to evaluate the benefit of Vemurafenib in colorectal cancers.

## 1. Introduction

Colorectal cancer is the third most commonly diagnosed cancer and leading cause of cancer death in men and women. In 2017, 1688780 new cancer cases and 600920 cancer deaths are projected to occur in United States [1]. Activating mutations in human Kirsten rat sarcoma viral oncogene (KRAS) are detected in approximately 40 percent of metastatic colorectal cancer (mCRC). Mutations in murine sarcoma viral oncogene homolog B1 (BRAF) are found in 5 to 10 percent of mCRC [2].

The RAS-RAF-MEK-ERK signaling pathway (MAPK pathway) is a classical intracellular pathway that plays a crucial role in homeostasis of normal cell turnover, cellular proliferation, differentiation, survival, and apoptosis. The activation of the epidermal growth factor receptor (EGFR) signaling cascade is a well-known pathway that can lead to colon cancer. Mutation within the KRAS exon 2 and extended RAS (i.e., KRAS exons 3 and 4 and NRAS exons 2, 3, and 4) oncogene located downstream to EGFR within this pathway leads to its activation, even if the EGFR is blocked [3–5]. Consequently, tumors with mutated KRAS and probably BRAF are resistant to anti-EGFR therapy [6]. CRYSTAL, PEAK, and PRIME are few of the most important randomized controlled trials that have demonstrated that addition of anti-EGFR drugs to chemotherapy in patients with KRAS mutations has detrimental outcomes [7–9]. Here, we report a case of metastatic colon cancer with coexistent KRAS and BRAF mutations. Concomitant KRAS and BRAF mutant CRCs are rare, occurring in less than 0.001% of cases [10].

## 2. Case Presentation

A 29-year-old noncigarette smoking Hispanic woman with no significant medical, family, and surgical history presented to the clinic with complaints of abdominal pain, nausea, fatigue, constipation, and subjective fevers at nights for 3 weeks. Vital signs were stable and physical exam was remarkable for mild tenderness in left lower quadrant. Laboratory tests indicated the presence of microcytic hypochromic iron deficiency anemia and mild elevation in her liver function enzymes. Hemoglobin was 7.8 and AST/ALT/ALP were 92, 34, and 144. Computerized tomography (CT) scan showed a large pelvic mass (11 cm) with uterine mass (fistulous communication to colon), retroperitoneal, pelvic lymphadenopathy, and widespread hepatic metastasis. The fevers, abdominal pain, and constipation were attributed to her malignancy and the uterine mass was thought to be a fibroid.

Follow-up positron emission tomography scan (PET scan) was obtained to look for primary malignancy and it showed abdominal, pelvic lymphadenopathy and innumerable necrotic lesions in the liver concerning for metastasis and a pelvic mass. As her imaging raised suspicions for malignancy, she underwent liver biopsy and flexible sigmoidoscopy. Biopsy of the hepatic lesions showed adenocarcinoma and immunohistochemical staining was positive for pan-cytokeratin, CMA5.2, villin, and CDX2 and negative for PAX- 8, ER, PAX-5, CK7, CK20, Hep-par1, and vimentin. FISH (fluorescent in situ hybridization) for high risk HPV was found to be negative. Immunohistochemical staining was suggestive of GI (gastrointestinal) primary or a carcinoma with enteric differentiation. Tumor markers were as follows: CA19-9 = 981, CA-125 = 205, and CEA = 284.3. Flexible sigmoidoscopy showed circumferential necrotic mass which was present 11 cm from the anal verge extending 7-8 cm distally. Mass was very friable with bleeding noted even with minor scope friction. Biopsies were obtained and they were sent for extended RAS mutation analysis as colorectal cancer was thought to be primary based on the workup obtained so far. Biopsies were also tested for mutations and they were positive for KRAS mutation (C 35 G>A/p. G12D) and BRAF mutation exon 15 codon 600 at V600E (GAG), V600D (GAT), or V600K (AAG). Her pathologic staging was pT4N1M1.

She was not a good candidate for surgical management, so decision to start chemotherapy was made. As she was found to be positive for both KRAS and BRAF, she was not considered a candidate for anti-EGFR treatment. Anti-VEGF treatment (Bevacizumab) could not be considered in her case as she had an active infectious fistulous communication between a uterine fibroid and the bowel wall. FOLFOX therapy was administered for 2 cycles and she tolerated it well. Further cycles of chemotherapy were not administered as she was deemed unresponsive to treatment based on visualization of progressive lesions and poor performance status. Unfortunately, her disease progressed rapidly and she expired within 3 months from the time of her first diagnosis.

## 3. Discussion

This rare case of metastatic colorectal cancer with concomitant KRAS and BRAF mutations had several unusual features including young age of presentation with no risk factors, harboring concomitant KRAS and BRAF, and rapid progression of disease to death within 3 months of the onset of disease. This case report emphasizes the importance of obtaining baseline testing of KRAS and BRAF mutations as it appears that the patients who have coexistent mutations tend to have aggressive course of illness as evident in this patient as she survived only 3 months from the time of diagnosis. Possible mechanism of having coexistent KRAS and BRAF mutation is unknown as its frequency is very low and it is not clear whether or not these tumors have a different biology and natural history than KRAS or BRAF mutant tumors or which of the two mutations is the dominant oncogene driving tumor proliferation [8]. A study by Seth et al. has shown that coexistent KRAS and BRAF tumor mutations have been observed in 1 cell line of the 24 cell lines that have been studied but information on clinical impact was not available. Also we do not know if KRAS or BRAF or both are driver mutations [11]. Another possible mechanism to be considered is the presence of biclonal population of tumor cells, with one clone harboring KRAS mutation and the other clone harboring BRAF mutation.

Epidermal growth factor receptor (EGFR) targeted antibodies were approved for clinical use in patients with metastatic CRC. Several retrospective analyses of prospective clinical trials in CRC patients receiving anti-EGFR antibody treatment have shown that patients with mutated KRAS did not benefit from anti-EGFR therapy. The KRAS data has changed the paradigm of anti-EGFR antibody treatment in CRC. CRYSTAL and PRIME are two most important phase III randomized controlled trials that have demonstrated that addition of anti-EGFR drugs to chemotherapy has detrimental outcomes in KRAS mutant mCRC.* CRYSTAL trial* studied FOLFIRI +/− Cetuximab in mCRC. The median OS (overall survival) with FOLFIRI + Cetuximab was 24.9 months in wild type KRAS and 17.5 months in mutant KRAS. The* PRIME* study (FOLFOX4 +/− Panitumumab) showed median OS of 23.9 months in wild type KRAS and 15.5 in mutant KRAS.* PEAK* study is the first randomized trial which studied the effect of Panitumumab (anti-EGFR antibody) + mFOLFOX6 compared to Bevacizumab (anti-VEGF antibody) + mFOLFOX6 for first-line treatment of wild type KRAS metastatic colon cancer. PFS was similar but OS was statistically and clinically improved in Panitumumab arm (median OS 41.3 months) when compared to Bevacizumab arm (median OS 28.9 months). In the prospective secondary analysis of PEAK study, patients with extended RAS wild type were found to have PFS of 13 months in Panitumumab arm and 9.5 months in Bevacizumab arm [7].

The impact of BRAF has also been retrospectively evaluated on tissue from completed prospective trials. MRC (Medical Research Council) COIN trail was the largest trial that studied the effect of anti-EGFR treatment (Cetuximab) to chemotherapy regimen of Oxaliplatin and fluoropyrimidine in mCRC; and the effect was further analyzed for KRAS, NRAS, and BRAF mutations. This was the first trial that showed that addition of anti-EGFR drugs to chemotherapy in BRAF mutant mCRC had worse outcomes. Median overall survival was shorter in BRAF mutant CRC (8.8 months) than in those with BRAF and KRAS wild type tumors (17.5 months) [12].

Vemurafenib is FDA approved to be used in BRAF mutant melanoma. Single agent Vemurafenib which is an inhibitor of BRAF V600E has been tested in BRAF mutant CRC but failed to show antitumor activity [13]. When Vemurafenib blocks BRAF activity and cuts off signaling within the MAPK pathway, this kicks on a feedback mechanism leading to upregulation of upstream EGFR, once again driving signaling through the MAPK pathway upon which these tumors are so dependent. To prevent this molecular escape route, the addition of EGFR inhibitor Cetuximab was tested in a clinical trial conducted by Southwest Oncology group (NCT02164916). 99 patients with BRAFV600E-mutated and RAS wild type mCRC received irinotecan and Cetuximab +/− Vemurafenib. The addition of Vemurafenib to irinotecan and Cetuximab doubled the median PFS from 2.0 months to 4.4 months. Of note, none of these patients were KRAS mutant. So we still do not know the optimal management for metastatic CRC harboring concomitant KRAS + BRAF mutations.

## 4. Conclusions

Many centers test for KRAS mutation. If it is found to be KRAS mutant, then BRAF testing is not performed. However, if KRAS is wild type, then BRAF testing is obtained in a sequential manner. On the basis of this case experience and review of literature, we conclude that mCRC harboring concomitant KRAS + BRAF mutations is a distinct and highly aggressive subset which is in dire need of new therapeutic strategies. Hence, baseline testing of KRAS, extended RAS, and BRAF mutations needs to be obtained as a standard of care in the clinical management of mCRC patients instead of sequential testing approach. We are currently conducting a retrospective chart review study to assess the incidence and biology of coexisting mutations in mCRC at University of Kansas Medical Center. In addition, we will evaluate coexisting RAS (KRAS and extended RAS) and BRAF mutations as a prognostic marker and as a tool to predict a more aggressive biological phenotype of metastatic colon cancer. Metastatic CRC with concomitant RAS + BRAF mutations should be assigned to a separate arm in clinical trials to evaluate the role of novel therapeutics for this deadly disease.
